# Bridging the connection between effective viscosity and electrical conductivity through water content in the upper mantle

**DOI:** 10.1038/s41598-018-20250-2

**Published:** 2018-01-29

**Authors:** Yixian Xu, Anqi Zhang, Bo Yang, Xuewei Bao, Qinyan Wang, Jianghai Xia, Wencai Yang

**Affiliations:** 1 School of Earth Sciences, Zhejiang University, Hangzhou, 310027 China; 2 Institute of Geophysics and Geomatics, China University of Geosciences, Wuhan, 430074 China

## Abstract

Upper mantle viscosity plays a key role in understanding plate tectonics and is usually extrapolated from laboratory-based creep measurements of upper mantle conditions or constrained by modeling geodetic and post-seismic observations. At present, an effective method to obtain a high-resolution viscosity structure is still lacking. Recently, a promising estimation of effective viscosity was obtained from a transform derived from the results of magnetotelluric imaging. Here, we build a relationship between effective viscosity and electrical conductivity in the upper mantle using water content. The contribution of water content to the effective viscosity is isolated in a flow law with reference to relatively dry conditions in the upper mantle. The proposed transform is robust and has been verified by application to data synthesized from an intraoceanic subduction zone model. We then apply the method to transform an electrical conductivity cross-section across the Yangtze block and the North China Craton. The results show that the effective viscosity structure coincides well with that estimated from other independent datasets at depths of 40 to 80 km but differs slightly at depths of 100 to 200 km. We briefly discussed the potentials and associated problems for application.

## Introduction

In multi-scale geodynamic modeling^1–3^, the investigation of fine-scale deformation in the lithosphere^4,5^ and interpretation of large-scale geophysical data^6–8^, the spatial variation of effective viscosity plays a key role. The effective viscosity of the lithospheric mantle depends on the composition, differential stress, ambient temperature and pressure, water and its fugacity, the grain sizes of minerals, and so on. In light of many geophysical imaging results, the regional-scale heterogeneity is naturally decreased with depth, and the heterogeneity in the depths of <200 km is fundamentally an ubiquitous feature^9^. This fact strongly demands a high-resolution estimation of effective viscosity for lithospheric dynamics. The up-to-date methods for estimation of effective viscosity distribution in an upper mantle may comprise a minimum of extrapolation from laboratory-based measurements^10^, modeling of geodetic measurements of postglacial rebound and/or post-seismic deformation^11^. The reliability of laboratory-based data exploration is doubtful^10^. The modeling of geodetic measurements is obviously lacking high resolution and generally fails for the case with lamellar decoupling in an upper mantle. It is thus particularly attractive for a method that may get an estimation of effective viscosity from a high-resolution geophysical imaging.

In the presumption of constant strain rate, the effective viscosity is mainly affected by water content, temperature and pressure. Compared to the estimate of water content, the temperature and pressure can be more easily constrained. On the other hand, the electrical conductivity is also affected mainly by temperature and water content in the upper mantle^12^, except for the occurrence of melts^13–18^.

Recently, a plausible connection between the effective viscosity and electrical conductivity had been demonstrated in the western United States at depths of 40–200 km^19^. This pioneering study creates a means of imaging the effective viscosity of the upper mantle with a high spatial resolution. However, the parameters of the transform of electrical conductivity to effective viscosity, such as the exponent index and pre-exponent coefficient, are determined by incorporating observational constraints that include surface topography and intraplate deformation^19^. This scheme is thus lacking a direct physical basis for the relationship between the effective viscosity and electrical conductivity.

It is widely accepted that the Moho temperature is the most dominant parameter in controlling the integrated lithospheric strength^20^. A heat shielding effect in crust caused by laminated structure of mafic-to-ultramafic intrusions may lead to a biased estimation of the Moho temperature^21^. This effect can be identified by seismic reflections and velocity model. The extreme lateral variation of temperature in the lithospheric mantle should generally be within 300 *K* and is expected to be minor in an intraplate environment^22^. We hence propose that the error associated with temperature estimation by current schemes^23,24^ causes a change of electrical conductivity that is smaller than one logarithmic unit. Conversely, the water content at the same depth in the upper mantle can change from dozens to more than 1000 ppm^14,25–28^, resulting in a variation of electrical conductivity of more than one logarithmic unit based on a simple calculation by using the recently calibrated power law relation^29^. The variation of effective viscosity in the same range of water content can change by a factor of 3 to 6^30^. It is thus clear that the water content is the most important factor controlling both the electrical conductivity and effective viscosity, which motivates us to build a clear physical relationship between them by incorporating the water content as the connecting parameter.

## Method

A low water content limit in olivine in a given range of upper mantle temperature and pressure defines the ‘dry’ condition here and constructs an upper limit of effective viscosity at a constant strain rate. We define the effective viscosities for the ‘dry’ (*i* = 0) and ‘wet’ (*i* = *w*) upper mantle, respectively, for a constant strain rate $({\dot{{\rm{\varepsilon }}}}_{0})$ as follows

1 $${\eta }_{i}={({\dot{\varepsilon }}_{0})}^{(1-n)/n}{[{A}_{i}{C}_{i}^{r}exp(-\frac{{H}_{0}-\beta {C}_{i}^{1/3}}{RT})]}^{-1/n},\,$$

where *A*_*i*_ is the pre-exponential constant for water content at *C*_*i*_; *r* and *n* are the exponents of the water content and stress, respectively; *β* is the constant coefficient; *H*_0_ is the activation enthalpy for the dry olivine aggregates (${H}_{0}={E}_{0}+P{V}_{0}$, where *P* is pressure, and *E*_0_ and *V*_0_ are the corresponding activation energy and volume, respectively); *R* is the ideal gas constant; and *T* is the absolute temperature. Clearly, the contribution of water content to the effective viscosity has been isolated in eq. (1). The ‘wet’ condition thus represents all cases for water content that are greater than *C*_0_ in the upper mantle. Then, we can get the following

2 $$\frac{{\eta }_{w}}{{\eta }_{0}}={[\frac{{A}_{w}}{{A}_{0}}\cdot \frac{{C}_{w}^{r}}{{C}_{0}^{r}}]}^{-1/n}exp[-\frac{\beta ({C}_{w}^{\frac{1}{3}}-{C}_{0}^{\frac{1}{3}})}{nRT}].\,$$

Traditionally, the electrical conductivities in the ‘dry’ (*i* = 0) and ‘wet’ (*i* = *w*) conditions are obtained by only considering the proton conduction with a pre-exponential constant ${\sigma }_{p}$ and an activation energy *H*_*p*_, which can be defined as follows:

3 $${\sigma }_{i}={\sigma }_{p}{C}_{i}^{{r}_{e}}exp(-\frac{{H}_{p}-\alpha {C}_{i}^{1/3}}{RT}),$$

which results in

4 $$\frac{{\rho }_{w}}{{\rho }_{0}}=\frac{{\sigma }_{0}}{{\sigma }_{w}}=\frac{{C}_{0}^{{r}_{e}}}{{C}_{w}^{{r}_{e}}}exp(-\frac{\alpha ({C}_{w}^{\frac{1}{3}}-{C}_{0}^{\frac{1}{3}})}{RT}),\,$$

where ${\rho }_{w}$ and ${\rho }_{0}$ denote ‘wet’ and ‘dry’ resistivities, respectively.

Due to the similarity between eqs (2) and (4), we assume the ratio of viscosity defined in eq. (2) is proportional to the ratio of resistivity defined in eq. (4), i.e.,

5 $$\frac{{\eta }_{w}}{{\eta }_{0}}={b}_{0}{(\frac{{\rho }_{w}}{{\rho }_{0}})}^{{b}_{1}},$$

we obtain

6 $${b}_{0}{(\frac{{C}_{w}}{{C}_{0}})}^{-{r}_{e}{b}_{1}}{[exp(-\frac{\alpha ({C}_{w}^{\frac{1}{3}}-{C}_{0}^{\frac{1}{3}})}{RT})]}^{{b}_{1}}={(\frac{{A}_{w}}{{A}_{0}})}^{-1/n}{(\frac{{C}_{w}}{{C}_{0}})}^{-r/n}exp[-\frac{\beta ({C}_{w}^{\frac{1}{3}}-{C}_{0}^{\frac{1}{3}})}{nRT}].\,$$

Equation (6) has three unknowns: *b*_0_, *b*_1_, and *β*. Supposing ${b}_{0}={(\frac{{A}_{w}}{{A}_{0}})}^{-1/n}=11.66$ (*A*_0_ = 1258925.412 ${(MPa)}^{-n}{s}^{-1}$, *A*_*w*_ = 794.3282 ${(MPa)}^{-(n+1.2)}{s}^{-1}$)^31^, ${r}_{e}{b}_{1}=r/n$, and $\beta =n\alpha {b}_{1}$. By choosing *n* = 3 and $r=1.2$^31,32^, we immediately get three sets of ${b}_{1}$ and *β* for electrical conduction models derived through the fitting of the experimental data^29,33,34^, as shown in Table 1.

**Table 1:** The parameters β and *b*_1_ determined by different electrical conductivity relations.

Laboratory-based relation	r_e_	*α*	β	*b*_1_
		${{\boldsymbol{\sigma }}}_{{\bf{Y}}}$ and ${{\boldsymbol{\sigma }}}_{{\bf{J}}}$ in eV/wt %; ${{\boldsymbol{\sigma }}}_{{\bf{G}}}$ in kJ/mol/wt ppm^1/3^		
${{\rm{\sigma }}}_{{\rm{Y}}}$ ^(^^34^^)^	1	0.16 ± 0.02	0.192	0.4
${{\rm{\sigma }}}_{{\rm{G}}}$ ^(^^19^^)^	1	2.08 ± 0.55	2.496	0.4
${{\rm{\sigma }}}_{{\rm{J}}}$ ^(^^33^^)^	0.86	0.09 ± 0.08	0.126	0.465

It is worth noting that the determined parameters are specific but not optimal solutions. We note that Liu and Hasterok^19^ chose ${b}_{0}=1$ and ${b}_{1}=0.6667$ (personal communication) in the transformation of resistivity to effective viscosity. In the present scheme, the reference viscosity corresponds to a ‘dry’ upper mantle, which is different from the regionally averaged viscosity used in ref.^19^.

**Figure 1:**
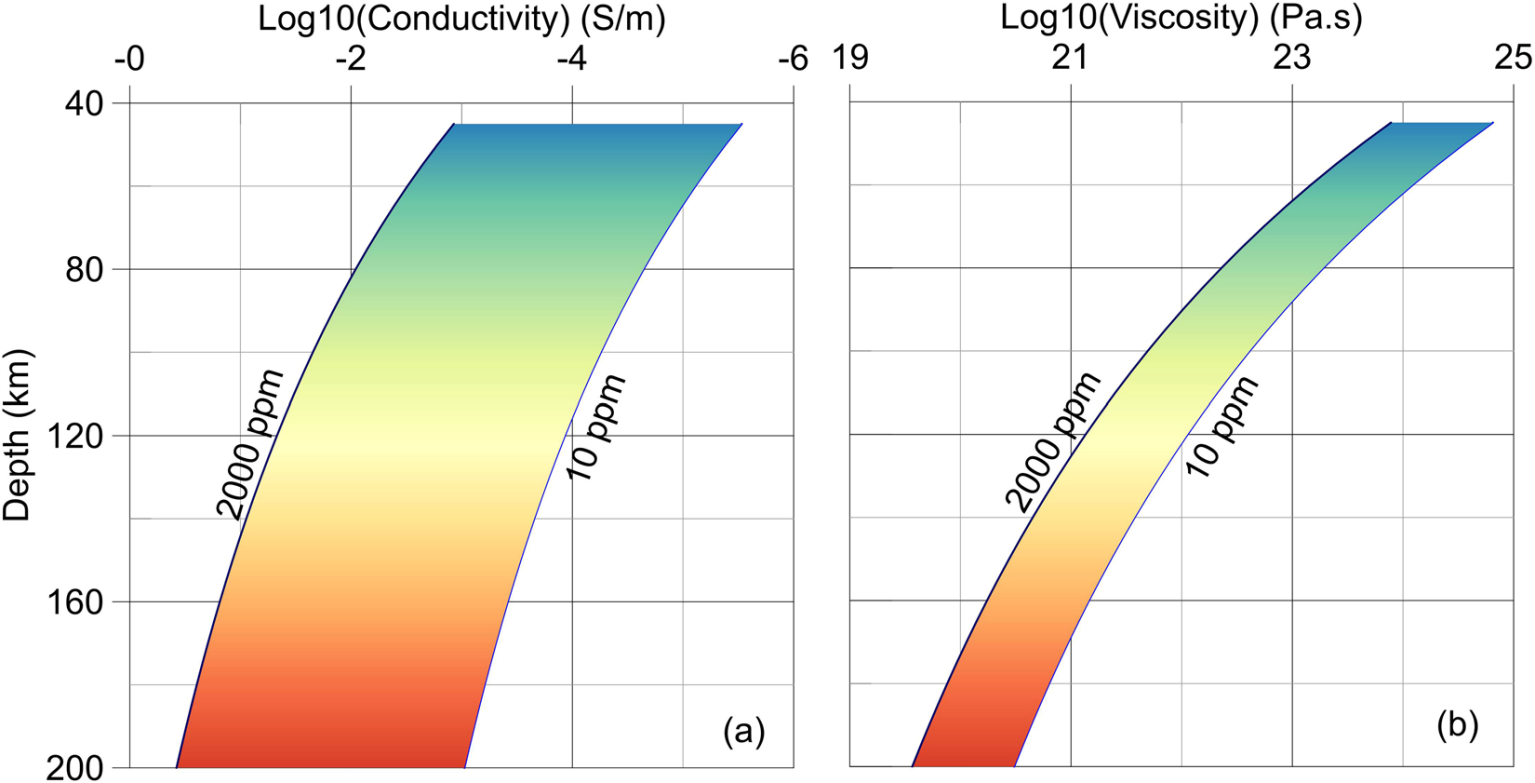
Variation of electrical conductivity (**a**) and effective viscosity (**b**) with depth for various water contents (10 to 2000 ppm) through the Gardés relationship^19^. The color is proportional to depth and thus, temperature.

According to the effective viscosity and electrical conductivity models, variations with depth (and thus, temperature) have been calculated and are shown in Fig. 1. Here, it is assumed that the Moho discontinuity is located at a depth of 40 km, with a temperature of 823 K and a crustal density of 2800 kg/m^3^. In addition, the geothermal gradient and density in the upper mantle are assumed to be 4.8 K/km and 3200 kg/m^3^, respectively. The constant strain rate is assumed to be 10^−14^ s^−1^. The electrical conductivities are calculated by using the recently calibrated relationship, which was derived from all available experimental data covering the broad ranges of temperature and water content^29^. As illustrated in Fig. 1, the electrical conductivity is much more sensitive to the change of water content than is the effective viscosity in the upper mantle, indicating that the prediction of effective viscosity from electrical conductivity is rather robust. On the other hand, compared to the electrical conductivity, the viscosity seems to be more sensitive to temperature, which is more significant in a low-temperature domain, i.e., in the uppermost part of the mantle.

## Examples

### Intraoceanic Subduction Zone

**Figure 2:**
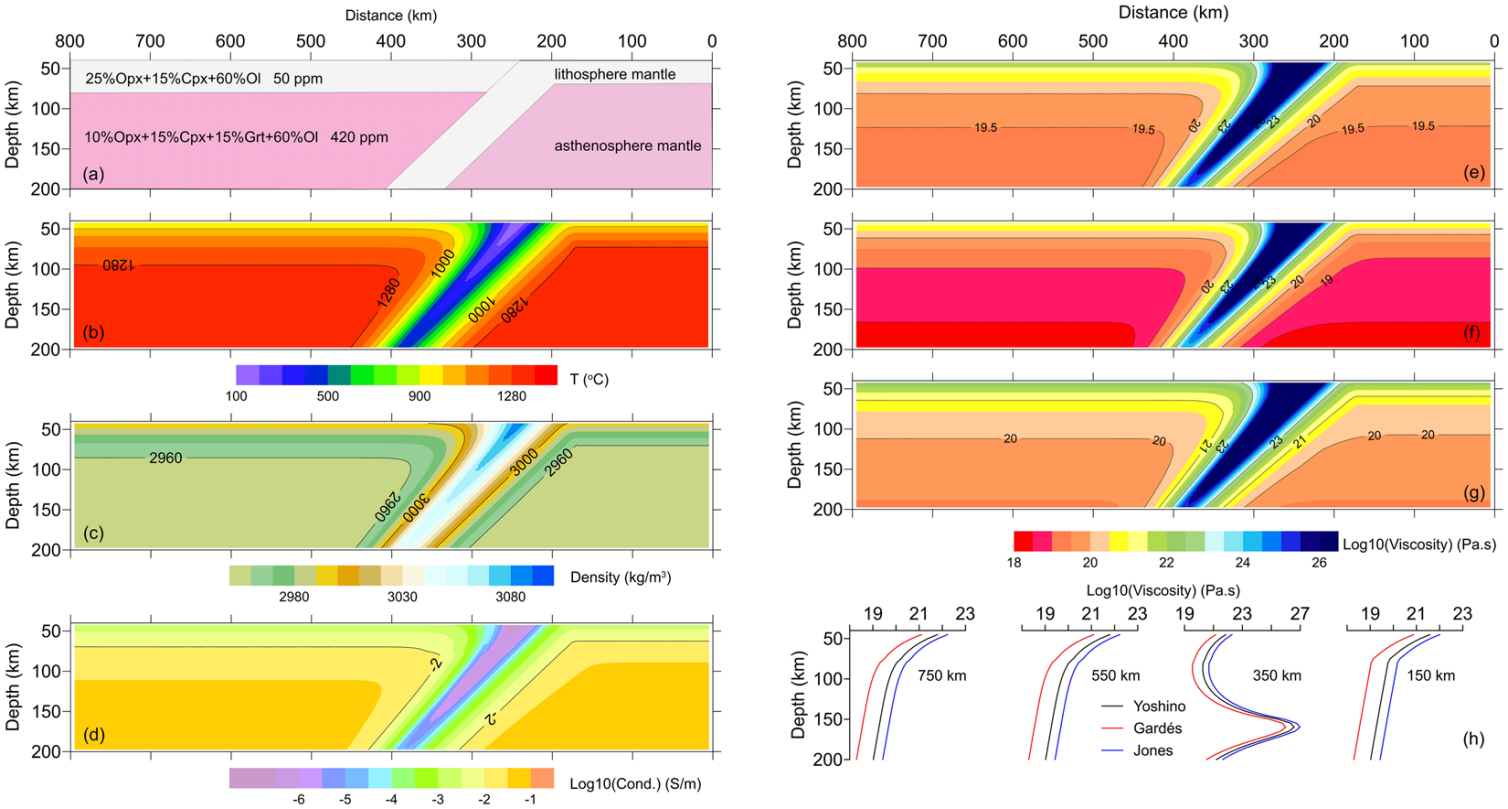
Distributions of composition (**a**), temperature (**b**), density (**c**) and electrical conductivity (**d**) for a presumed subduction zone at depths of 40 to 200 km. The composition of the asthenospheric mantle has 15% more Grt and 15% less Opx than the lithospheric mantle. The water content changes from 50 ppm in the lithospheric mantle to 420 ppm in the asthenospheric mantle. The temperature and density distributions are modeled by TEMSPOL^24^. The electrical conductivity distribution is calculated by a previously proposed method^38^ and described in the text; the effective viscosities in (**e**) to (**g**) are transformed from eq. (5) with the parameters shown in Table 1; (**h**) shows the curves for effective viscosity versus depth at distances of 150, 350, 550 and 750 km along the model surface. Note that it is plausible that the three calibrated relations are inappropriate in a low-temperature domain (<300 °C), which may promote the effective viscosity in the interior of subduction plate.

To demonstrate the rationality of our scheme, we designed a simple model of an intraoceanic subduction zone with compositional geometry at depths of 40 to 200 km (Fig. 2(a)). This model represents a developing intraoceanic subduction zone without consideration of partial melting anywhere. The original oceanic lithosphere is presumed to be a single layer with an age of 15 Ma and to be underlain by a homogeneous asthenospheric mantle. The lithospheric thicknesses are 70 and 75 km for the overriding and subducting plates, respectively. The choice coincides a global mean (70 ± 4 km) determined by receiver function imaging of Ps converted phases at various oceanic island stations^35^. The temperature distribution prior to subduction is calculated from the GDH1 model^36^. After 10 Ma of subduction, the distributions of temperature (Fig. 2(b)) and density (Fig. 2(c)) are calculated by TEMSPOL^24^. We use different experimental relations to calculate the electrical conductivities of single-crystal minerals: the Yoshino relation^33^ for olivine (Ol) and garnet (Grt), and the Xu relationship^37^ for clinopyroxene (Cpx) and orthopyroxene (Opx). Then, we use an effective medium theory to produce a self-consistent solution of electrical conductivity in the upper mantle^38^. The laboratory-based electrical conductivity profile has been constructed for the specific model (Fig. 2(d)). Finally, the effective viscosities (Fig. 2(e–g)) are estimated by using eq. (5) with the parameters listed in Table 1.

The geometries of the three resulting viscosity models display very similar features, revealing that the effective viscosity decreases with depth in the upper mantle on both sides of the subducting plate. The effective viscosity within the subducted plate is approximately 3–6 orders larger than that of the ambient upper mantle, which guarantees a sustainable subduction. However, among the three transformed viscosity models, there are considerable discrepancies: the results from the Yoshino relation (Fig. 2(e)) are ~0.4 log units less than that of the Jones relation, on average (Fig. 2(g)), and ~0.7 log units larger than that of the Gardés relation (Fig. 2(f)). The results coincide with the analyses based on the sensitivity of electrical conductivity to the water content^33^. The effective viscosity of asthenosphere mantle (10^18^~10^20^ Pa.s) is well overlapped with results ((0.5–10) × 10^18^ Pa.s) constrained by post-seismic deformation in Indian Ocean^11^ and various estimations in western US^30^.

### A Magnetotelluric Transect from the Yangtze Block to the Southern North China Craton

**Figure 3:**
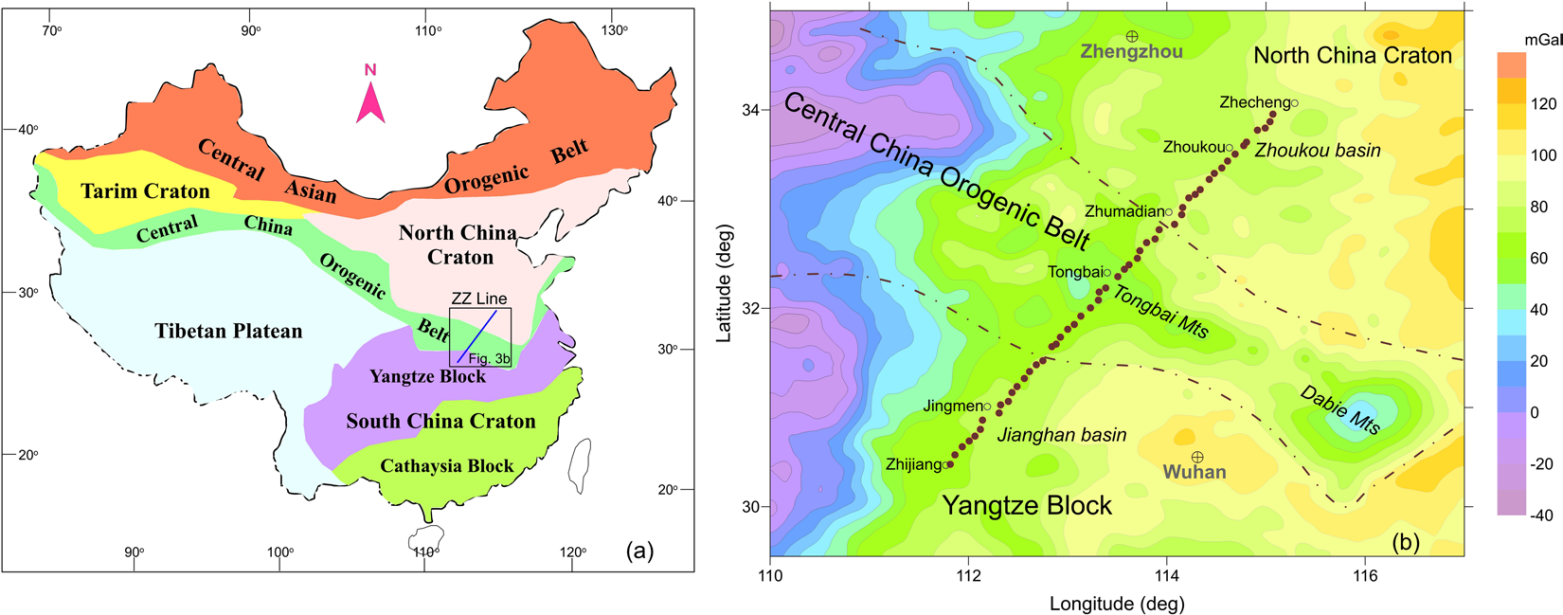
(**a**) MT transect line of Zhijiang-to-Zhecheng (ZZ) on a simplified tectonic map (generated by hand drawing in Grapher 10); and (**b**) 51 deployed MT stations (solid circles) on a regional free-air anomaly map of satellite gravity; the dot-dashed lines represent the sutures among the North China Craton, Central China Orogenic Belt and the Yangtze Block.

**Figure 4:**
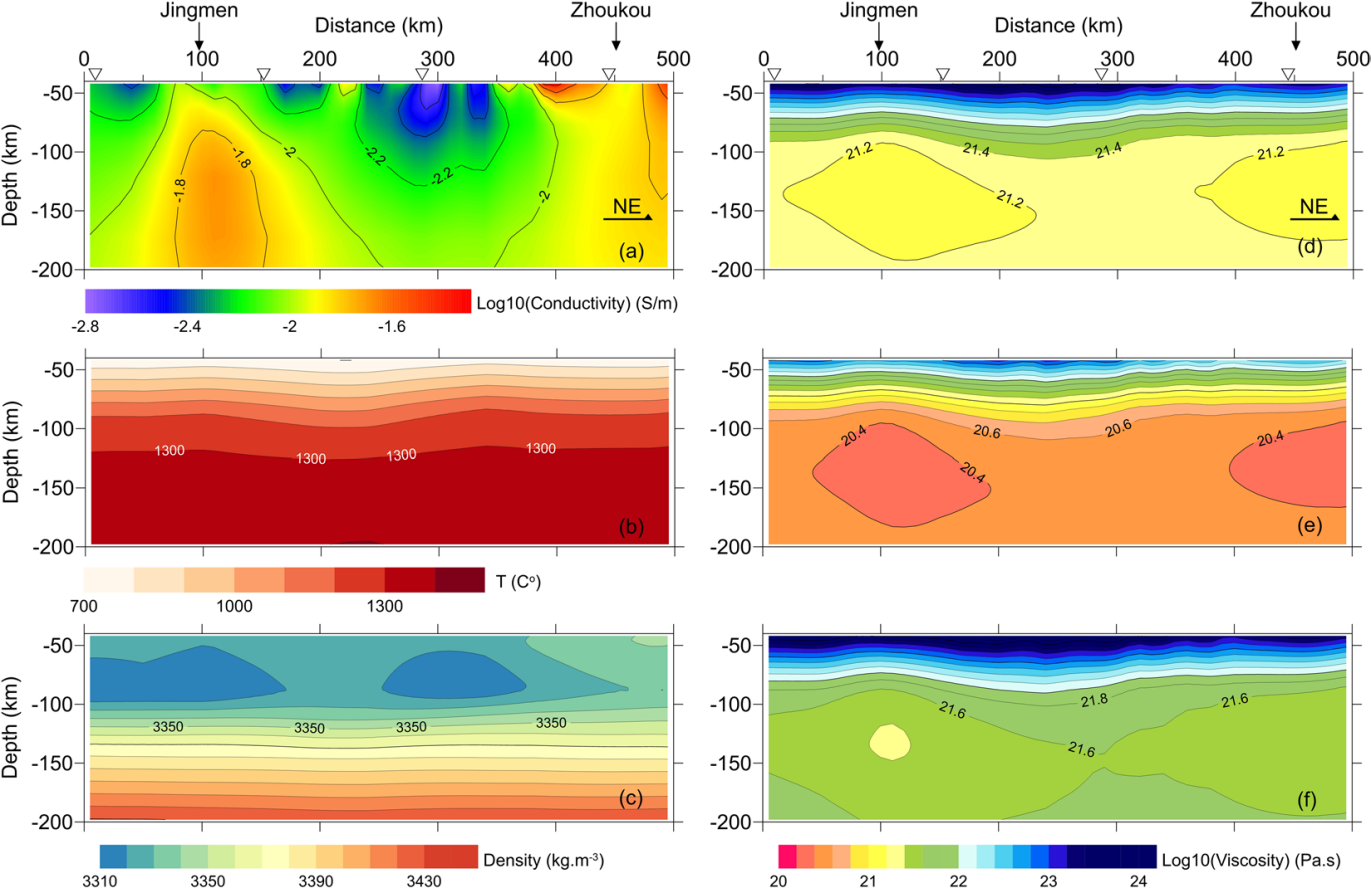
(**a**) Electrical conductivity cross-section; (**b**) and (**c**) are temperature and density, respectively, and are extracted from the results inverted from the Rayleigh wave dispersion, surface heat flow, geoid height and topography by the Bayesian inference approach^24,42,43^. The effective viscosities in (**d**) to (**f**) are transformed from eq. (5) with the associated parameters in Table 1. The four triangles in (**a**) and (**d**) mark the locations for comparison in Fig. 5.

The transect line ZZ, delineated in Fig. 3(a), from Zhijiang in the Yangtze block to Zhecheng in the North China Craton consists of 51 magnetotelluric stations (Fig. 3(b)). The cross-section of electrical conductivity (Fig. 4(a)) is extracted from the results inverted by a 3-D scheme using a non‒linear conjugate gradient (NLCG) algorithm^39^ in the ModEM computational framework^40,41^, in which the full MT impedances and tippers are used in periods of 396 Hz to 5000 seconds. The distributions of temperature and density (Fig. 4(b) and (c)) are modeled from Rayleigh wave dispersion curves, surface heat flow, geoid height and topography by a Bayesian inference approach^23,42,43^. The minimum water content is 10 ppm, based on geochemical analyses of mantle inclusions in eastern China^28^, and the strain rate from the GPS measurement^44^ is ${10}^{-15}{{\rm{s}}}^{-1}$, which has been used previously in China continents^45^. The transformed effective viscosities from eq. (5) with the associated parameters in Table 1 for the Yoshino, Gardés and Jones relations are shown in Fig. 4(d)–(f).

**Figure 5:**
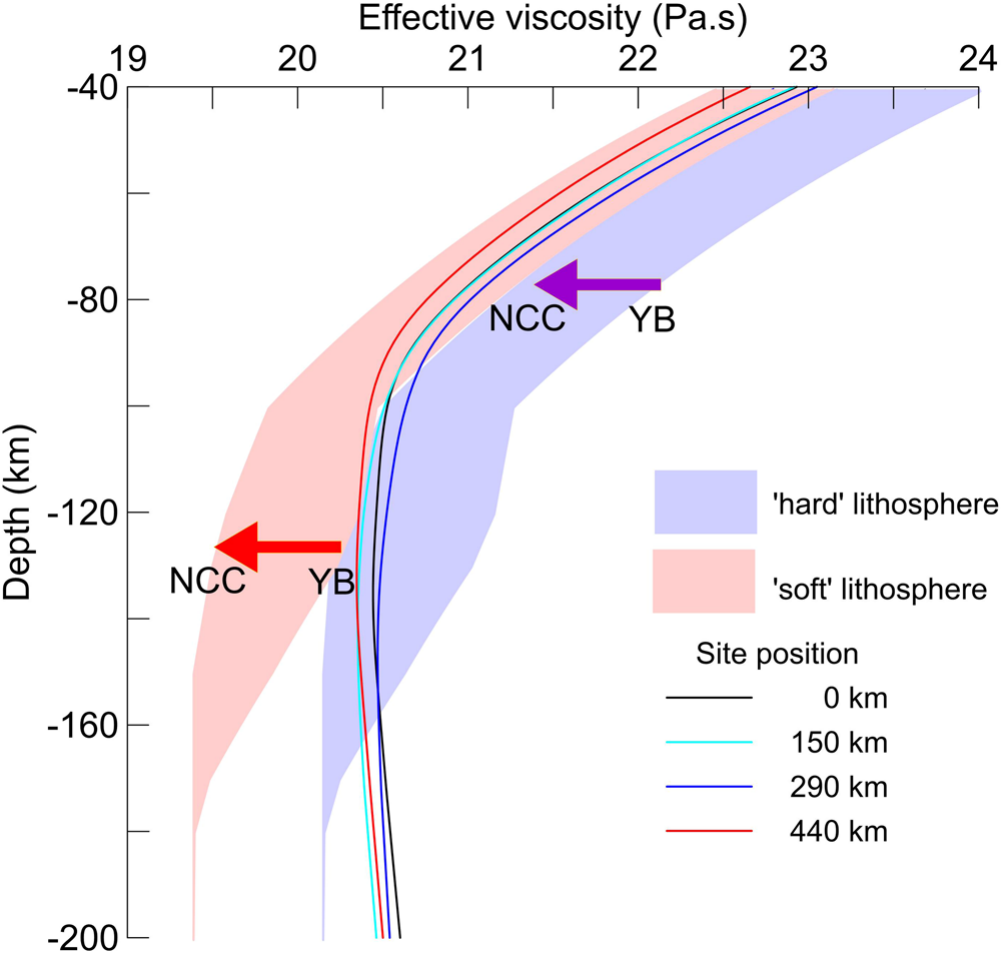
Comparison of the effective viscosities from the present study (solid lines for the Gardés relation) and ref.^45^ (shadowed powder blue and faded pink for ‘hard’ and ‘soft’ lithosphere, respectively) at four sites along the MT transect. The site positions along the MT transect are marked in colors. The purple and red arrows indicate regular decreases from the northern Yangtze block (YB) to the southern North China Craton (NCC) for the ‘hard’ and ‘soft’ lithosphere^45^, respectively.

Regardless of the differences between the three profiles, a strong and thick lithosphere is clearly observed beneath the Central China Orogenic Belt (Tongbai Mountains), and the lithosphere beneath the north margin of the Yangtze block is slightly thicker than that beneath the southern North China Craton (Fig. 4(d–f)). Moreover, there are two weak zones in the upper mantle beneath Jingmen and Zhoukou (Fig. 4(d–f)), associated with the Jianghan and Zhoukou Basins (Fig. 3(b)), coinciding with their regional extensions during the Cenozoic era.

To evaluate the reliability of the transformed effective viscosities, we compare our result with that from ref.^45^, in which they combined a crustal thermal model^46^ and an upper mantle thermal structure^47^, and assigned a ‘soft’ or ‘hard’ rheology to the lithospheric layers^45^. Due to the low spatial resolution of their results, we compare two independent datasets at only four sites along the MT transect. The possibility of partial melting can be excluded in the region because the inverted electrical conductivity along the transect is unlikely to indicate that the melts exceed the threshold of 0.5 vol% to form a significant conduction phase in the upper mantle^18,48^.

As illustrated in Fig. 5, our results clearly coincide with those derived from a ‘soft’ lithosphere at depths of <100 km and with those derived from a ‘hard’ lithosphere at depths of >100 km; the transition depth of the decreasing trend is at the depth of ~80 km. The range and decreasing trend of effective viscosities between two independent results are the same up to ~80 km. The effective viscosities from ref.^45^ continuously decrease at depths of 80 to 180 km. However, our results show only a slow decrease at the depths of 80~150 km followed by a gentle increase at depths of 150–200 km. The difference is unlikely to be caused by a small polaron conduction and ionic conduction at higher temperatures^49^ because neglecting these conduction mechanisms would increase the contribution of proton conduction (water) to electrical conductivity and cause an underestimation of the effective viscosity, according to eq. (5). If there is a mid-lithosphere discontinuity (MLD) at depths of 80–100 km in the region, it is plausible that the estimation of effective viscosity, based on a pure diffusion creep as we have used in the transformation, will cause the deviation. This is because a MLD has been reported as a seismic discontinuity and may represent a transition from a pure diffusion creep domain to a dislocation creep dominated domain^50^ or an elastically accommodated grain-boundary sliding (EAGBS) domain^12,51,52^. However, this transition of deformation mechanisms will generate mechanical decoupling and cause a biased estimation for the scheme used in ref.^45^ as well. In other words, if the lithosphere is not entirely mechanically coherent, the presumption of constant strain rate is not applicable to the whole domain no matter what method is used. Another significant difference is that the maximum effective viscosity is beneath the Tongbai Mountains (Fig. 5), rather than the north Yangtze block^45^. Considering the very weak seismicity in this region and the nearly symmetrical free-air gravity on both sides of the Tongbai Mountains (Fig. 3(b)), we have a good reason to justify the present results, i.e., the lithospheric root persists beneath the orogen, and the lithosphere thins with increasing distance from the orogenic center.

## Discussion and Conclusions

We present a scheme to transform an electrical conductivity model to an effective viscosity distribution in the upper mantle, where the effective viscosity and electrical conductivity are connected by isolating the contribution of water content in the flow law. Our method is based on the physical connection rather than on an empirical relation. Compared with the transformation scheme proposed in ref.^19^, an additional improvement in the result of our method is the selection of a reference effective viscosity that corresponds to a ‘dry’ upper mantle, which is clearer in physics and can be performed easily. The reliability of our method has been demonstrated by a synthetic model and a real-world example.

It is noteworthy that the present scheme cannot directly apply to a melt interconnecting domain, which can be understood as the domain that basaltic melt fraction is larger than 0.5% in volume. This value has been proposed as a melt interconnectivity threshold and loosely associated with bulk conductivity of 0.1 S/m for the melt-bearing olivine aggregate^18^ and used in the upper mantle beneath the southern Canadian Cordillera^53^. Actually some empirical models^18,54^ can be incorporated in our approach to convert melt conductivity into viscosity for a specific melt composition, however, both of the melt fraction and composition are thought rather hard to determine today. Additionally few of the laboratory-based conductivity measurements and the super-long-period MT data can be used for depth more than 10 GPa, which prevents us to discuss the efficiency of our approach in deeper upper mantle.

Realistically, the uncertainties of our essentially laboratory-based method must be carefully evaluated. First, the reliability depends upon the precision of measurement-calibrated relations for both the effective viscosity and electrical conductivity. Two demonstrations have shown that the uncertainties can go beyond one log unit for the Yoshino, Gardés and Jones calibrated relations; this is expected to improve continuously with the growing number of high-temperature and high-pressure measurements^29^. Second, as we cannot experimentally constrain the time- and/or size-dependent geological effects on rheological properties, it is difficult to estimate the uncertainty caused by any calibrated flow law for mantle rocks. Additional uncertainties could result from an electrical conductivity model produced by the inversion of magnetotelluric data. The third aspect of uncertainty comes from an estimation of mantle composition, which has been a long debated topic in petrology and geochemistry^55–58^. Finally, electrical anisotropy in the upper mantle composition^16,59,60^ must be addressed as well. This phenomenon may indicate the rheological anisotropy and is still poorly known^51^.

In summary, regardless of the uncertainties, which cannot be completely resolved in the proposed procedures and related formulations, our method can robustly constrain an effective viscosity distribution with a resolution as high as that of MT imaging in the upper mantle.
